# The Disruption of Memory Consolidation of Duration Introduces Noise While Lengthening the Long-Term Memory Representation of Time in Humans

**DOI:** 10.3389/fpsyg.2019.00745

**Published:** 2019-04-03

**Authors:** Joffrey Derouet, Valérie Doyère, Sylvie Droit-Volet

**Affiliations:** ^1^CNRS, UMR 6024, Laboratoire de Psychologie Sociale et Cognitive, Université Clermont Auvergne, Clermont-Ferrand, France; ^2^CNRS, UMR 9197, Institut des Neurosciences Paris-Saclay, Université Paris-Sud – Université Paris-Saclay, Orsay, France

**Keywords:** timing, time, long-term memory, memory consolidation, memory

## Abstract

This study examined the effect of an interference task on the consolidation of duration in long-term memory. In a temporal generalization task, the participants performed a learning phase with a reference duration that either was, or was not, followed 30 min later by a 15-min interference task. They were then given a memory test, 24 h later. Using different participant groups, several reference durations were examined, from several hundred milliseconds (600 ms) to several seconds (2.5, 4, and 8 s). The results showed that the scalar timing property (i.e., precision proportional to judged duration) was preserved despite the interference task given during the memory consolidation process. However, the interference task increased the variability of time judgment and tended to produce a lengthening effect in all reference duration conditions. The modeling of individual data with parameters derived from scalar expectancy theory suggests that disrupting the memory consolidation of learned reference durations introduces noise in their representation in memory, with time being specifically distorted toward a lengthened duration.

## Introduction

More than 120 years ago, Müller and Pilzecker (1900) proposed the “perseveration-consolidation” hypothesis for the stabilization in long-term memory of newly learned information (Lechner et al., 1999; McGaugh, 2000; Hernandez and Abel, 2008). Their pioneering laboratory studies showed that electroconvulsive shock (ECS) delivered between 15 and 60 min after learning produced deficits in memory retention, and that this deficit decreased as the learning-ECS interval increased (for a review, see Glickman, 1961). These studies therefore confirmed that newly acquired information is unstable, but consolidates over time. It has since been found that injecting a specific drug (puromycin, a protein synthesis inhibitor) in goldfish affects retention scores in long-term memory (4 days after learning), but not those in “short-term” memory (at the day of learning) (Agranoff et al., 1965). Different mechanisms therefore underlie short-term memory and long-term memory. As previously found with ECS, the disruption of long-term memory weakens as the interval between the training and the time at which the treatment (injection) is applied lengthens. This finding shows that the stabilization of new memories in long-term memory involves specific molecular processes that take time. The time-course of the molecular mechanisms responsible for memory consolidation depends on the type of learning, ranging from one hour to several hours (McGaugh, 2002).

The consolidation process in long-term memory has been examined in humans for various types of tasks and information. For example, a modulation of consolidation has been demonstrated in perceptual learning of auditory discriminative features (Maidment et al., 2015) or after incidental learning of visual objects (Borota et al., 2014), as well as in learning of motor skills (Walker et al., 2003). The aim of the present study was to examine the effect of a disruption of memory consolidation processes on long-term judgments of duration in humans.

Only two studies in humans have experimentally examined the consolidation of duration in long-term memory (Rattat and Droit-Volet, 2010; Cocenas-Silva et al., 2014). These studies used a generalization task because, in that task, the participants must refer to a memory representation of the learned duration to judge the similarity between that duration and probe durations. In a learning phase, the participant initially learns an unique reference duration. Then, in a testing phase, he/she judges whether or not the probe duration is the same as the previously learned reference duration. Rattat and Droit-Volet (2010) compared the effect of a retention interval of 15 min and 24 h between the learning phase of a reference duration of 4 s and the testing phase on temporal judgment, and did not find any difference in temporal generalization gradients peaking around 4 s. This is consistent with an accurate representation of time in long-term memory when the storage of time in memory is not disrupted. However, when an interference task was introduced immediately after the learning phase, the subjects tended to judge the reference duration as being shorter than it actually was. In addition, their time estimates were more variable with the interference task than without. However, in that study, the interference task was given immediately after learning the reference duration, leaving the possibility that the shortening of time could have resulted from a disruptive retroactive effect on the storage of time in short-term working memory and/or its manipulation in working memory, rather than from a specific consolidation process in long-term memory. Indeed, a shortening effect was observed in animals when a short-term retention interval (5–20 s) was introduced between the response and the stimulus duration presentation (e.g., Church, 1980; Spetch and Wilkie, 1983; Spetch, 1987; Grant and Spetch, 1993; Spetch and Grant, 1993). A similar shortening effect was found in humans when a short-interval was introduced between the reference duration and its subsequent testing (Wearden and Ferrara, 1993; Wearden et al., 2002).

To examine the consolidation process of time in long-term memory, Cocenas-Silva et al. (2014) thus decided to introduce a 15-min interference task not just after the learning phase of the reference duration (4 s), but 30 min afterward. Moreover, they tested the memory of reference duration either immediately after the interference task or after a 24-h delay. In these conditions, they found that the interference task distorted the time judgment, with memory for durations tending to be shortened (underestimated) in the immediate test (consistently with Rattat and Droit-Volet’s finding) and lengthened (overestimated) in the delayed test. Indeed, the generalization gradient shifted toward the left with the interference task compared to no interference task in the short-term condition, while it shifted toward the right in the long-term condition, peaking at longer temporal values. The generalization gradient was also flatter (greater variability in temporal judgment) in the interference than in the non-interference condition for the long-term test (24 h), while they did not differ between these two conditions for the immediate test. These differences in temporal performance are in line with the existence of different mechanisms for the memory storage of duration in the short and long term. In addition, a temporal gradient of the effect of interference was observed in the long-term condition (24 h after learning). Indeed, the temporal bias and the variability in time judgment in the long term decreased when the interval between learning and the interference task was increased from 30 min to 1 h. This finding is consistent with the idea that the consolidation of newly acquired information in long-term memory is time-dependent.

The study conducted by Cocenas-Silva et al. (2014) was the first to obtain results consistent with a specific long-term consolidation process for memory traces of stimulus duration in humans. However, they used only one reference duration (4 s) that makes it impossible to generalize their results to other shorter or longer durations. The aim of the present study was thus to examine the effect of a disruption of consolidation process in long-term memory of time with other reference durations, i.e., from the sub-second to supra-second range, using Cocenas-Silva et al. (2014) paradigm. In addition, as they discussed, it is not known whether the temporal lengthening effect observed in long-term memory is due to a distortion of the reference duration in memory or simply to a decisional bias in temporal judgment. In the temporal generalization task, the gradient is usually symmetrical in animals (Church and Gibbon, 1982) and right-skewed in humans (Wearden, 1992). Church et al. (1991) thus concluded that the right asymmetry in the generalization gradient is not linked to timing processes *per se*, but to a decision rule independent of time processing. As explained by Wearden (2004, page 4), “the asymmetry in the generalization gradient is not a product of internal clock processes, nor of the way times are remembered.” Recently, Lamotte et al. (2017) showed that humans adopted low conservative decision strategies because they were overconfident in their temporal responses in the generalization task. It is therefore possible for participants to change their decision strategies at the second test day or/and because they are aware of the decrease in the quality of their long-term memory time representation after an interference task. To try to identify the mechanisms underlying the temporal generalization judgment in a 24-h delayed test, we modeled our data using the SET-based model (Gibbon, 1977,^1991^; Gibbon et al., 1984) that was initially developed by Church and Gibbon (1982) for animals’ responses on the generalization task and then modified by Wearden (1992) for humans. This model, fully described in the results below, tested three parameters: a decisional parameter and two memory parameters. One memory parameter addresses the distortion in the representation of the reference duration, and the other the variability (noise) in its representation.

In the present study, we therefore tested participants using a generalization task involving the learning of a reference duration and a test deferred by 24 h, comparing in different groups a wide range of reference stimulus durations, from milliseconds (600 ms) to several seconds (2.5, 4, and 8 s). For each reference duration, two conditions were examined in independent sub-groups: with and without a 15-min interference task presented 30 min after the learning of the reference duration. Our hypotheses were that the disruption of the memory consolidation process by an interference task would produce a lengthening effect and increase the variability of time judgment in the temporal generalization task at all time scales. The modeling of our data should indicate whether the effects mainly result from time distortion in the memory representation of the reference duration and/or to a noisier temporal representation.

## Materials and Methods

### Participants

A total of 200 undergraduate psychology students at Clermont Auvergne University (Mean age = 19.66, *SD* = 1.62 years) participated in this experiment in return for course credits. They filled in the consent form describing the experimental procedure, which was reviewed and approved by the Sud-Est VI Statutory Ethics committee (CPP).

### Material

The experiment took place in a small soundproof room. The visual temporal stimulus was a gray disk (2.5 cm in diameter) displayed at the center of the computer screen. An E-prime program (2.0. Psychology Software Tools) controlled all experimental events and recorded the data. The “D” and “K” keys of the computer keyboard were used for the subject’s responses: “yes” and “no,” respectively. To start any trial in the learning and the test phases, the participant was instructed to press the “space bar” on the keyboard after the word “Prêt” (meaning “Ready”) had been displayed. For the interference task, the backward digit recall task from the Wechsler Memory Scale (WMS-II, Wechsler, 1998) was used, as in Cocenas-Silva et al. (2014). The participant was instructed to recall, in backward order, a series of digit sequences containing a number of digits that progressively increased from sequence to sequence (from 2 to 8 digits). There were two trials per sequence, and a total of seven sequences that were administered repeatedly during a 15-min period.

### Procedure

The participants were randomly assigned to one of four groups (50 participants per group) for training in a temporal generalization task with different reference durations: 600 ms, 2.5, 4, and 8 s. In each duration group, the participants were first trained (learning phase) and then tested 24 h later (test phase). In the learning phase, the participant had to learn and memorize the reference stimulus duration: The disk cue was first presented 5 times for the reference duration. This was followed by two training blocks of four trials each, including two trials with the reference stimulus duration (600 ms, 2.5, 4, or 8 s) and one trial with each of two easy comparison durations (200 and 1000 ms, 0.313 and 4.688 s, 0.5 and 7.5 s, and 1 and 15 s, respectively). The inter-trial interval was randomly selected between 0.5 and 1.0 s. During the training phase, the participant was instructed to respond whether the presented duration was or was not identical to the reference duration by pressing the corresponding key, “yes” or “no.” “Correct” or “wrong” feedback was given after each response. The participants were explicitly told not to count during the presentation of the stimuli because this can bias scientific results (for the efficacy of methods used to prevent counting, see Rattat and Droit-Volet, 2012). In addition, as we can see below, our data did not show a violation of the temporal scalar property characteristic of a counting effect on timing.

In the test phase, performed 24 h after the learning phase, 72 trials composed of 8 blocks of 9 random trials were presented. The nine trials consisted of three trials with the reference duration (600 ms, 2.5, 4, or 8 s) and one trial with each of six other durations, chosen to be proportionally equidistant with respect to the reference duration for the different duration groups. The values were therefore as follows: 225, 350, 475, 725, 850, and 975 ms; 0.625, 1.250, 1.875, 3.125, 3.750, and 4.375 s; 1, 2, 3, 5, 6, and 7 s; 2, 4, 6, 10, 12, and 14 s, respectively. The participants had to judge whether the stimulus duration was or was not the same as the reference duration they had learned the previous day. No feedback was given.

To examine the effects of disruption in the consolidation process on time judgment, two subgroups of participants were formed for each duration group: a group of participants that performed a 15-min interference task 30 min after the learning phase (Interference group), and a control group that did not perform any interference task (No-interference group) (see Cocenas-Silva et al., 2014).

## Results

### Data Analysis

**FIGURE 1:**
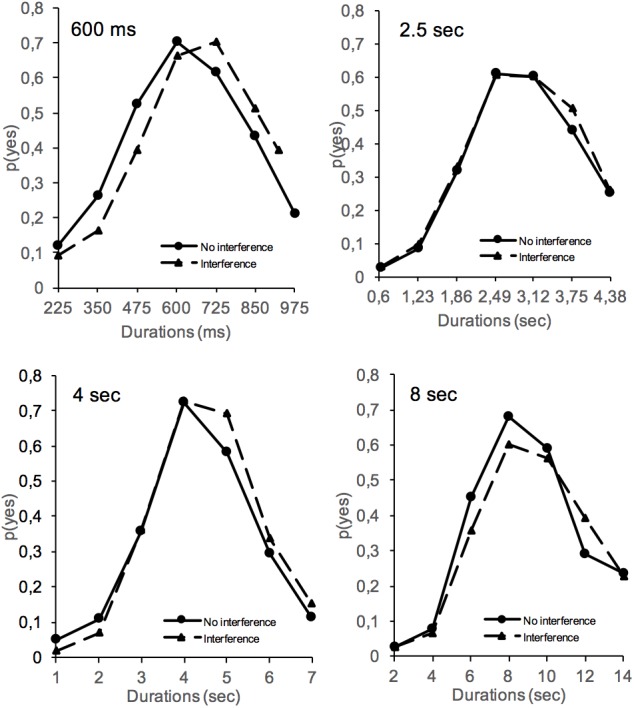
Proportion of “yes” responses (meaning “it is the reference duration”) obtained in the long-term memory test (24 h after acquisition) plotted against stimulus durations for the 600-ms, 2.5-s, 4-s, and 8-s reference duration groups with (dashed lines) and without (solid lines) an interference task during the long-term memory consolidation process.

**FIGURE 2:**
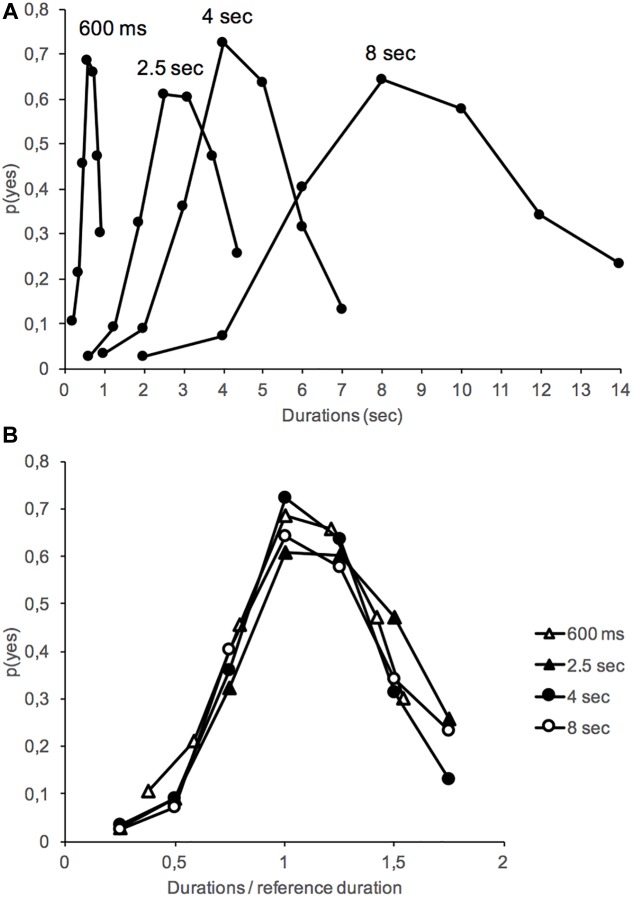
Mean proportion of “yes” responses obtained in the long-term memory test (24 h after acquisition) plotted against absolute (**A** top) or relative (**B** bottom) stimulus durations for the 600-ms, 2.5-s, 4-s, and 8-s duration groups.

Figure 1 shows the proportion of “yes” responses – *p*(yes) – plotted against stimulus duration in the interference and no-interference conditions for the different duration groups. An analysis of variance (ANOVA) was performed on *p*(yes) with two between-subjects factors (interference and duration groups) and one within-subjects factor (stimulus duration tested). A Greenhouse–Geisser correction was used for the different repeated-measures ANOVAs when a violation of sphericity was observed. The ANOVA on *p*(yes) showed a significant main effect of stimulus duration, *F*(6,1152) = 205.25, *p* < 0.001, and of duration group, *F*(3,192) = 11.95, *p* < 0.001, with a significant stimulus duration × duration group interaction, *F*(18,1152) = 2.01, *p* = 0.007. As illustrated in Figure 2A, this interaction suggests a flattening of the generalization gradient as the reference duration to be judged increased. However, the generalization gradients superimposed well when the proportion of “yes” responses was plotted as a function of each stimulus duration expressed as a fraction of the reference duration (Figure 2B). This superposition of generalization gradients with a rescaled time axis is the hallmark of the scalar property of time judgment (Gibbon, 1991; Wearden and Lejeune, 2008). This property indicates that the standard deviation (SD) of temporal responses is a linear function of their mean, such “as timing measures superimpose when plotted on the same relative scale” (Wearden, 2016, p. 33). The scalar property of time was therefore maintained in long-term memory, i.e., when the judgment was performed 24 h after the learning of a reference duration.

Importantly, the ANOVA also yielded a significant interaction between the interference condition and the stimulus duration, *F*(6,1158) = 2.27, *p* = 0.03, which subsumed neither a significant main effect of interference, *F*(1,192) = 0.268, *p* = 0.605, nor any other significant interaction involving the interference factor (all *ps* > 0.05). Parsing the interaction, the analysis indicated that the participants responded “yes” more often in conditions with than without interference for stimulus durations longer than the reference duration [averaging on the 3 longest stimulus durations, 0.445 vs. 0.388, *F*(1,198) = 3.77, *p* = 0.05], but not for those shorter than [averaging on the 3 shortest stimulus durations, 0.167 vs. 0.20, *F*(1, 198) = 2.16, *p* = 0.14] or equal to [0.65 vs. 0.679, *F*(1,198) = 0.91, *p* = 0.34] the reference duration (Figure 1). The magnitude of the difference in *p*(yes) between the longest and the shortest durations was indeed larger with than without interference [0.278 vs. 0.187, *F*(1,198) = 3.355, *p* = 0.038]. This suggests a trend toward a temporal lengthening effect due to the interference task.

**FIGURE 3:**
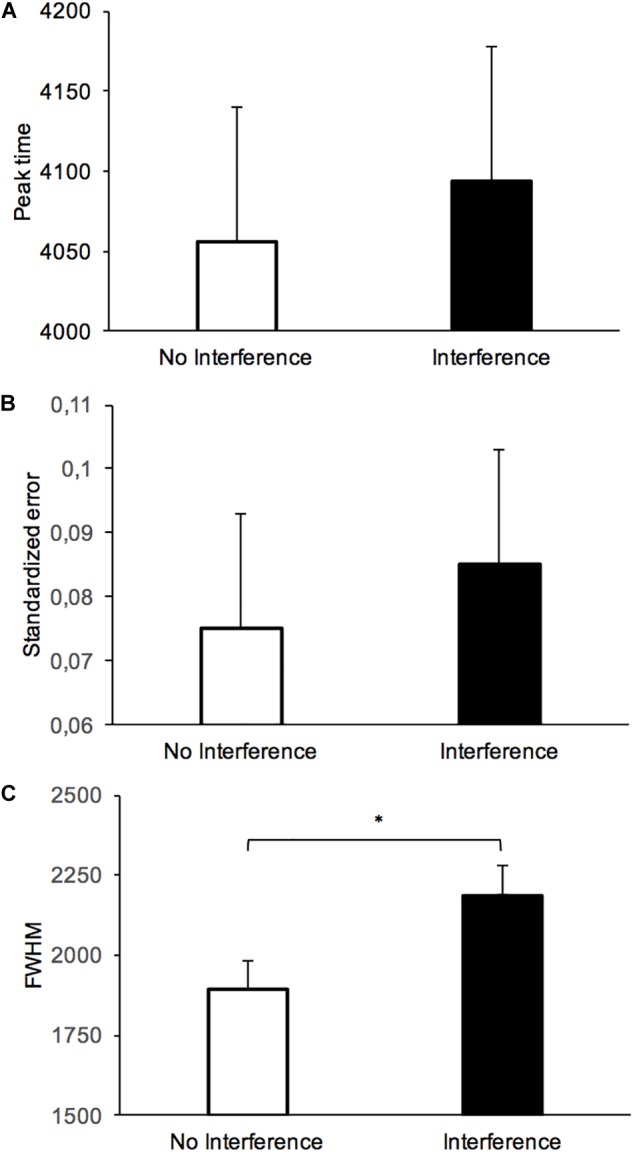
Mean (+*SD*) peak time (ms) **(A)**, standardized error **(B)**, and FWHM **(C)** in the long-term memory test with or without an interference task during the long-term memory consolidation process.

To further characterize the impact of the interference task on the variability and the accuracy of long-term temporal judgment, we calculated (a) the peak time, (b) the standardized error, and (c) the width of the temporal generalization gradient at half of its maximum height (full width at half maximum, FWHM) (Figure 3). The peak time is the stimulus duration corresponding to the highest proportion of “yes” responses. It is a measure of the stimulus durations judged similar to the reference duration. The standardized error is the difference between the peak time and the reference duration divided by the reference duration. This is thus a measure of the distance between peak time and reference duration in the direction of either an under- (<0.0) or over-estimation (>0.0). The FWHM is a measure of temporal variability. The peak time and the FWHM were obtained by fitting each participant’s generalization gradient with the “log normal (amplitude)” function from the PeakFit program (PeakFit Version 4.12). This procedure produced good fits of temporal gradients for most of the participants (mean *R*^2^ = 0.89, *SD* = 0.14). Table 1 shows the mean and standard error for the different measures obtained in the different groups.

**Table 1:** Mean (*SD*) Peak Time, Standardized Error and FWHM (variability) for the 24-h delayed test with and without an interfering task during the long-term memory consolidation process.

		Peak time		Standardized error		FWHM	
				
		M	*SD*	M	*SD*	M	*SD*
600 ms	No interference	610	*91*	0.02	*0.15*	287	*109*
	Interference	647	*91*	0.08	*0.15*	281	*134*
2.5 s	No interference	2752	*571*	0.10	*0.23*	1225	*439*
	Interference	2680	*578*	0.07	*0.23*	1261	*425*
4 s	No interference	4590	*710*	0.15	*0.17*	1970	*392*
	Interference	4481	*689*	0.12	*0.17*	2380	*721*
8 s	No interference	8272	*1542*	0.03	*0.19*	4093	*1391*
	Interference	8567	*1275*	0.07	*0.16*	4828	*1391*


**FIGURE 4:**
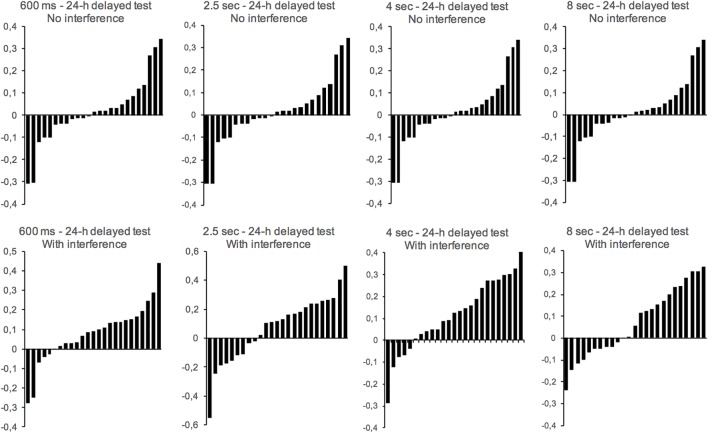
Distribution of individual standardized errors in the long-term memory test with (bottom panels) or without (top panels) an interference task for the 600-ms, 2.5-s, 4-s, and 8-s duration groups (one panel per group). Each bar represents one subject.

The individual peak time data, represented on Figure 4 using standardized errors, suggested a lengthening effect compatible with the one described above for the proportion of “yes” responses for the interference condition compared to the no-interference condition. However, in the delayed tests used in our study, there was a great inter-individual difference, such that the ANOVA conducted on the peak time with two between-subjects factors (interference and duration groups) showed a significant main effect of duration group, *F*(3,192) = 773.05, *p* < 0.001, but no main effect of interference, *F*(1,192) = 0.101, *p* = 0.75, and no interaction between interference and duration group, *F*(3,192) = 0.59, *p* = 0.62 (Figure 3A). For the standardized error (Figure 3B), there were also no significant effects (all *p* > 0.05).

**FIGURE 5:**
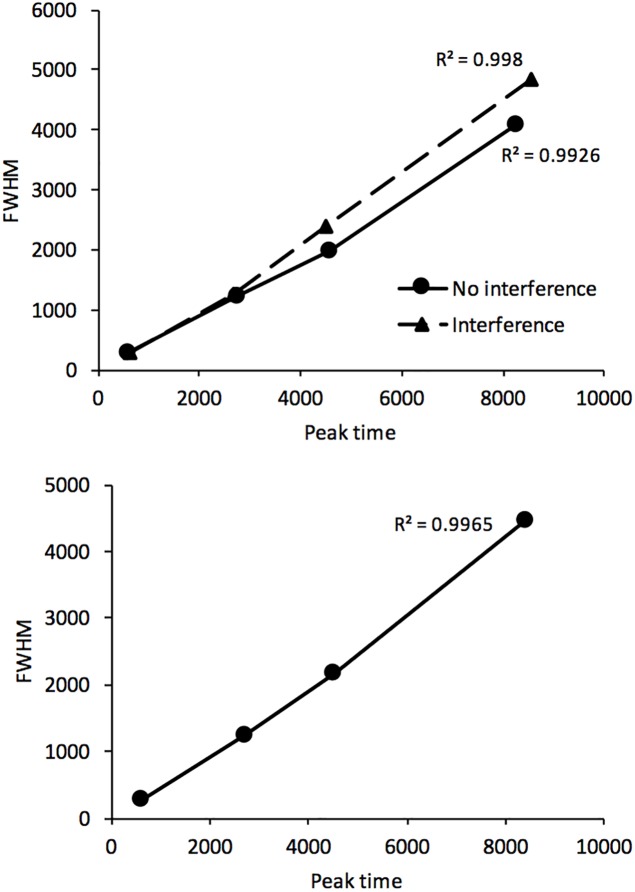
FWHM plotted against peak time in the long-term memory test with or without an interference task (top) or these two conditions combined (bottom).

By contrast, the ANOVA performed on the FWHM showed a clear significant main effect of interference, *F*(1,192) = 5.28, *p* = 0.023, with a main effect of duration group, *F*(3,192) = 195.81, *p* < 0.001, but no significant interference x duration group interaction, *F*(3,192) = 1.86, *p* = 0.14. Therefore, in agreement with the scalar property of time, the variability of temporal estimates increased linearly with the duration range even with a 24-h interval between learning and test (*R*^2^ = 0.9965, *p* < 0.0001) (Figure 5). More interestingly, whatever the duration range, the variability of time judgment was greater with (*M* = 2187.41, *SE* = 199.31) than without a task (*M* = 1893.81, *SE* = 159.47) assumed to be interfering with the consolidation process for durations in long-term memory (Figure 3C).

### Theoretical Modeling

We next attempted to identify the mechanisms underlying the higher temporal variability in long-term memory, and the trend toward a lengthening effect for *p*(yes), when an interference task is introduced after the learning phase. To do this, we applied the generalization model originally used by Church and Gibbon (1982) for rats (Church and Gibbon model) and subsequently modified for human adults (modified Church et al., 1991) and children (Droit-Volet et al., 2001; Droit-Volet, 2002). This MCG model is fully described in numerous manuscripts or books (Wearden, 2016), such as that by Delgado and Droit-Volet (2007) in which a figure illustrates the effect of manipulating one parameter, while the others are kept constant, on the generalization gradient. This model estimates three parameters, *c*, *K*, and *b*, as follows. The reference duration in memory follows a Gaussian distribution with a mean *s* and a coefficient of variation *c*. The parameter *c* is thus the coefficient of variation of the memory representation of the reference duration, *s*. For each trial, a value *s*^∗^ is randomly sampled from this distribution. The model assumes that the participants respond “yes” when ∣*(s*^∗^ *– t)/t*∣ < *b*. *t* is the probe duration presented in the test phase. As explained by Delgado and Droit-Volet (2007, page 12, Figure 5), “increasing *c* makes the reference memory fuzzier and flattens the generalization gradient, although few “yes” responses occur at the shortest stimulus durations.” The parameter *K* is a “distortion parameter” (multiplier) applied to the reference memory *s*^∗^: If *K* is 1.0, the reference duration will be remembered accurately, if *K* is >1.0, it will be remembered as longer than it really was, and if *K* is <1.0, it will be remembered as shorter than it was. The parameter *b* is a decision parameter. Increasing *b* makes the decision of responding “yes” less conservative, so that the system responds “yes” to probe durations that are more distant from the reference duration. This produces an overall increase in the proportion of “yes” responses, while the general shape of the gradient remains constant.

Using a computer program written in Visual Basic, we fitted the model to the individual data. The fit was good for most of our participants, with a small mean absolute difference (*mean MAD* = *0.048*, *Min* = *0.001*, *Max* = *0.14*, *SE = 0.003*) between the data on obtained “yes” responses and those of the fitted function divided by 7 (the number of data points), except in the case of 6 participants (3% of participants) who obtained a *MAD* > 0.15. Table 2 reports the groups’ mean and SD obtained for the MAD and the three parameters derived from the fits of the model for each condition. An ANOVA performed for each parameter with two factors (duration group and interference) did not find any significant effect on the decisional parameter, *b*, for interference, *F*(1,186) = 0.10, duration group, *F*(3,186) = 0.82, or their interaction, *F*(3,186) = 0.34, all *ps* > 0.05. However, the ANOVA showed a significant main interference effect for both the coefficient of variation of the memory representation of the reference duration, *c*, *F*(1,186) = 4.87, *p* = 0.03, and the memory distortion parameters, *K*, *F*(1,186) = 7.37, *p* = 0.01, with neither a duration group effect, nor a duration group × interference interaction being found (all *ps* > 0.05). This confirms that the memory representation of the reference duration was more variable, or noisier, with (*M* = 0.30, *SE* = 0.17) than without (*M* = 0.24, *SE* = 0.018) interference that disrupted the consolidation process in long-term memory. Our modeling also suggested a time distortion in the reference duration toward a temporal lengthening for the interference condition (*M* = 1.04, *SE* = 0.014) compared to the no-interference condition (*M* = 0.989, *SE* = 0.015). In fact, the *K*-value was significantly less than 1 without the interference task, *t*(94) = 69.63, *p* = 0.0001, while it was significantly greater than 1 for the same test with the interference task, *t*(98) = 70.94, *p* = 0.0001.

**Table 2:** Parameter values for the fits of the modified Church and Gibbon model with the individual data obtained in the 24-h delayed test with and without an interference task.

		c		K		b		MAD	
					
		M	*SD*	M	*SD*	M	*SD*	M	*SD*
600 ms	No interference	0.28	*0.15*	0.93	*0.13*	0.30	*0.08*	0.05	*0.04*
	Interference	0.25	*0.13*	1.07	*0.12*	0.29	*0.07*	0.05	*0.04*
2.5 s	No interference	0.24	*0.18*	1.04	*0.14*	0.29	*0.07*	0.05	*0.04*
	Interference	0.34	*0.24*	1.05	*0.17*	0.30	*0.07*	0.05	*0.04*
4 s	No interference	0.22	*0.09*	1.01	*0.13*	0.27	*0.07*	0.05	*0.04*
	Interference	0.27	*0.20*	1.03	*0.14*	0.28	*0.05*	0.04	*0.04*
8 s	No interference	0.23	*0.09*	0.97	*0.14*	0.28	*0.07*	0.05	*0.04*
	Interference	0.32	*0.21*	1.03	*0.15*	0.28	*0.06*	0.05	*0.04*
Mean	No interference	0.24	*0.13*	0.99	*0.14*	0.28	*0.07*	0.05	*0.04*
	Interference	0.30	*0.20*	1.04	*0.15*	0.29	*0.07*	0.05	*0.04*


*c, coefficient of variation of the memory representation of the reference duration; K, distortion of the memory representation of the reference duration. b, decisional threshold. MAD, mean absolute difference between the data points and the fitted function divided by 7.*

## Discussion

Our study examined time judgment in a generalization task with different reference durations ranging from a few hundred milliseconds to multiple seconds, when the participants had to compare probe durations with the reference duration learned 24 h earlier. This temporal comparison task requires participants to remember the reference duration stored in long-term memory. Our findings testing ranges of reference durations from 600 ms to 8 s indicate that a newly learned duration is well-remembered 24 h later regardless of the temporal scale, and that the fundamental scalar property of timing observed in numerous studies in short-term memory is preserved in long-term memory. Indeed, in the 24-h deferred test used in our study, the peak of the generalization gradient remained close to the reference duration for all durations tested, from 600 ms to 8 s. At the same time, the width of the generalization gradients increased with the duration range, and the generalization gradients superimposed well when plotted against durations expressed as a fraction of the reference duration. As stated by Gibbon (1991), these features are the “hallmarks of scalar timing.” Therefore, scalar timing is observed in humans not only for perception of duration but also for memory of duration. Our results are consistent with findings that have shown no difference in time judgment between an immediate and deferred judgment when the reference duration has been correctly encoded and stored in long-term memory (Ogden et al., 2008; Rattat and Droit-Volet, 2010). There is indeed evidence that animals can recall an interval duration learned by conditioning, either for 24 h after a single training session (Davis et al., 1989; Díaz-Mataix et al., 2013) or for several weeks or months after lengthy training, although with some loss in performance, especially when the retention interval extends over months (Gleitman and Bernheim, 1963; Campbell and Haroutunian, 1981; Lejeune, 1989; Soffié and Lejeune, 1991; Höhn et al., 2011). In one of our own studies, we also showed that young children are able to remember a specific action duration 6 months after learning it (Rattat and Droit-Volet, 2007).

Our study also examined the effect of an interference task, performed 30 min after the learning of a reference duration, on the remembering of that duration 24 h later. Our results show that the interference task disrupted the remembering of the reference duration in long-term memory, whether the memorized reference duration was in the milliseconds or multiple seconds range, as temporal performance was poorer in the 24-h test with than without an interference task. This finding is thus in line with the idea that recent memory traces for all durations, even the shortest ones, are unstable and are still not consolidated in long-term memory 30 min after being learned (Cocenas-Silva et al., 2014). Lewis et al. (2011) even showed an improvement in the memory of a motor rhythm after one night, which suggests that the consolidation of a learned rhythm also continues during sleep (sleep-dependent consolidation) (see also Ortiz and Wright, 2010; Bratzke et al., 2014; Chen et al., 2016). One of the novelties of our study lies in testing reference durations from 600 ms to 8 s. Our statistical analyses showed that the interference effect on temporal performance in long-term memory does not differ significantly across the different duration values. The effect of the disruption of consolidation process therefore seems to be similar within a large duration range despite the fact that different brain regions may be primarily involved in the processing of very short and long durations (Meck et al., 2008). This may thus suggest that neuroplasticity mechanisms (new protein synthesis) underlying the stabilization of memory for newly learned durations are similarly involved whatever the brain regions involved. Alternatively, it may rely on a single common structure, such as the hippocampus, which is also involved in memory for duration (Meck et al., 1984, 2013). In agreement with the “systems consolidation” view, the hippocampus may have an active function in the formation and the temporary storage of different new memories, including learned durations (McGaugh, 2000; Squire and Bayley, 2007; Hernandez and Abel, 2008). The identification of cerebral mechanisms and brain areas involved in the memory consolidation of duration will require specific experiments, which will likely be performed with animals.

In our study, the poorer performance in the 24-h deferred test in the interference groups resulted, for all reference durations tested, from a more variable time judgment, with a flatter generalization gradient and a larger FWHM. However, there was also a time distortion in the proportion of “yes” responses with a rightward shift of the generalization gradient, indicating that more of the long than the short probe durations were judged similar to the reference duration when an interference task had been administered after training than when it had not been. This effect, observed in *p*(yes) responses, was nevertheless not strong enough to be reflected in a significant shift in the peak of the generalization gradient or the standardized error. Overall, our results clearly replicate those found by Cocenas-Silva et al. (2014) with the 4-s duration, but indicate that the interference task has more robust long-term effects on the variability of time judgment than on time distortion.

Importantly, the simulation of the individual data with the model reinforces the conclusion in terms of a lengthening of duration in long-term memory. Indeed, the other novel aspect of our study lies in the simulation of the individual data with the modified Church and Gibbon model (Wearden, 1992). The fit of the individual data with this model was good for most of the participants. It also allowed us to ascertain that the difference in the shape of the generalization gradients between the interference conditions did not come from decision processes, since the parameter *b* remained constant over conditions. In other words, the interference-based differences in temporal performance (the rightward shift of the generalization gradient) observed in our study were not due to new decisional strategies adopted by the participants during the testing session.

By contrast, the modeling of our data allows us to suggest that memory mechanisms were responsible for performance differences between the interference conditions. The value of the first memory parameter, i.e., the *c* parameter, was indeed significantly higher in the interference than in the no-interference condition. According to the SET timing model, the reference duration is represented in the form of a distribution of values with a mean equal to the reference duration, when *K* is equal to 1.0, and a variance that increases as the square of the mean (Gibbon, 1991). The representation of time in reference memory is thus “inherently noisy” (Church and Gibbon, 1982, p. 116). Delgado and Droit-Volet (2007) demonstrated that introducing noise in the reference memory increased the coefficient of variation of the memory representation of the reference duration (*c* parameter), thus flattening the generalization gradient. Therefore, disrupting the consolidation of the newly learned duration by means of an interference task could introduce noise into the representation of this duration. The second memory parameter, the *K* parameter, is a memory distortion parameter, such that when *K* < 1.0, the reference duration is remembered as shorter that it really was, and when *K* > 1.0, it is remembered as longer than it was. In line with the results on *p*(yes), the *K*-value differed significantly between the interference conditions, being greater when the interference task was administered than when it was not. Our modeling thus tends to suggest that presenting an interference task shortly after training distorts the memorized duration in the direction of a lengthening effect.

The question remains of how the interference task produces this increased variability and this trend toward a lengthening effect during the formation of temporal traces in memory. According to the interference theory of forgetting, interferences disrupt memories, which consequently become more confused and distorted (Neath, 1998). In the framework of the SET model, we can hypothesize that interfering information would produce a “mixed” distribution in reference memory, thus increasing the noise in the representation of the reference duration. This is consistent with the mixed-memory model proposed by Penney et al. (1998) to explain the modality effect when both auditory and visual durations are presented within the same session. However, the question of whether time distortion will take the form of a lengthening or a shortening effect will depend on the temporal context encountered during the retention interval. Ogden et al. (2008) showed that the immediate learning of a second reference duration produced a rightward or leftward shift of the generalization gradient when the initial reference duration was shorter or longer, respectively, than the second duration. The interference task used in the present study is a classical working memory task that was chosen because it loads working memory and to extend the findings of Cocenas-Silva et al. (2014) study using the same methodology. However, it also requires the manipulation of digits. It might thus be possible that the nature of the interfering task influenced the direction of the time distortion. Therefore, the comparison with other interference tasks will be necessary in order to ascertain whether the disruption of the consolidation of duration in long-term memory always produces a lengthening effect, or whether it relates to the type of interfering task.

In addition, the individual data reveal a great inter-individual difference in the peak of the generalization gradient for the delayed test conditions used in our study, with some subjects underestimating and others overestimating the reference duration. The source of inter-individual differences in the peak time may be related to the variability in the activities of human participants performed during a 24-h retention interval, which are difficult, or even impossible, to control experimentally. Some of our participants may have perhaps undertaken activities that interfered less with the initial learning conducted in the laboratory than the others did. In particular, it is possible that some of them incidentally learned other durations, which were shorter or longer than the reference duration, during the retention interval while the memory of the initial reference duration was not yet fully stabilized. Other experiments will be necessary to clarify the impact of the temporal context, as well as the nature of the interference task, on the type of time distortion found. Several questions therefore remain to be solved in the field of research on the consolidation of duration in memory, i.e., an area just beginning to be experimentally examined in humans. Our results clearly demonstrate, however, that the disruption of the consolidation of duration in long-term memory produces noise in the memorized representation of time.

## Author Contributions

JD, VD, and SD-V conceived the experiments. JD collected the data, analyzed the data, and drafted the manuscript. SD-V and VD provided the critical revisions and approved the final version of the manuscript.

## Conflict of Interest Statement

The authors declare that the research was conducted in the absence of any commercial or financial relationships that could be construed as a potential conflict of interest.
